# Healthcare Practitioners’ Perceptions of mHealth Application Barriers: Challenges to Adoption and Strategies for Enhancing Digital Health Integration

**DOI:** 10.3390/healthcare13050494

**Published:** 2025-02-25

**Authors:** Haitham Alzghaibi

**Affiliations:** Department of Health Informatics, College of Applied Medical Sciences, Qassim University, Buraydah 52571, Saudi Arabia; halzghaibi@qu.edu.sa

**Keywords:** mHealth, Sehaty app, primary healthcare, Saudi Arabia, barriers to mHealth adoption, telehealth

## Abstract

Background: Mobile health (mHealth) applications have transformed healthcare delivery by enhancing accessibility, patient monitoring, and clinician communication. Despite these advantages, significant barriers hinder their adoption among healthcare practitioners, limiting their effectiveness in primary care settings. Understanding these barriers is crucial for optimizing mHealth integration into healthcare systems. Aim: This study examines healthcare practitioners’ perceptions of barriers to mHealth application adoption, with a focus on the Sehaty app (version 1.3) in Saudi Arabia. It aims to identify key challenges, assess their impact on user engagement and system efficiency, and provide insights for enhancing digital health implementation. Methods: A cross-sectional survey was conducted among 409 primary healthcare practitioners using the Sehaty app. The study employed a structured questionnaire assessing ten major barriers to mHealth adoption, including technical, usability, privacy, and integration challenges. Descriptive statistics, ANOVA, *t*-tests, and correlation analyses were performed to examine differences across demographic groups and relationships among identified barriers. Results: Findings revealed that technical and usability challenges were the most significant barriers, with system compatibility (Mean = 3.64), slow performance (Mean = 3.43), and excessive task complexity (Mean = 3.45) among the most cited issues. Training and support limitations (Mean = 3.28) and workflow integration difficulties (Mean = 3.24) further hindered adoption. Correlation analysis indicated weak interdependencies among barriers, suggesting that targeted interventions addressing specific concerns may be more effective. ANOVA results showed that digital literacy significantly influenced perceptions of communication barriers (*p* = 0.046), while gender differences in usability and productivity constraints were marginally significant. Conclusions: The study underscores the necessity for improved system interoperability, user-centered design, and enhanced technical support to promote mHealth adoption. Addressing these challenges through strategic policy initiatives and infrastructure improvements is essential for fostering a more integrated and effective digital healthcare ecosystem.

## 1. Introduction

Mobile health (mHealth) applications have emerged as revolutionary instruments in healthcare, providing patients enhanced access to medical information, monitoring capabilities, and connection with clinicians. Notwithstanding its potential, the implementation and continued utilization of mHealth applications encounter considerable barriers from the patients’ viewpoint. These constraints encompass technological, usability, budgetary, and psychological aspects, constraining the efficacy and accessibility of these devices.

A significant obstacle is the usability of mHealth applications. Numerous patients encounter challenges in navigating these applications due to intricate user interfaces or inadequate design considerations for varied user requirements. Creating an intuitive interface for mHealth applications is essential, particularly given the varied user demographic that include older patients who may lack technological proficiency. They encounter difficulties with app utilization owing to restricted technical literacy and physical impediments such as impaired vision or dexterity challenges [1,2]. Moreover, the time investment necessary to acquire proficiency in these applications may dissuade busy individuals or those lacking motivation [2].

The deficiency of digital literacy among patients is a significant concern. A significant number of persons are oblivious to the availability or advantages of mHealth applications, while others possess insufficient technical skills to utilize them proficiently [2,3]. This disparity is especially pronounced among older demographics and individuals in marginalized places where access to technology and education is restricted. In the absence of focused initiatives to enhance digital literacy, these groups continue to be marginalized from the advantages offered by mHealth applications. Concerns around privacy and security serve as substantial deterrents for numerous patients contemplating mHealth applications. Users frequently express concerns regarding illegal access to sensitive health information or the exploitation of their personal data. These issues are exacerbated for applications addressing stigmatized disorders like as mental health or HIV/AIDS, where breaches may result in social isolation or prejudice [2,4]. Furthermore, numerous mHealth applications exhibit inadequate privacy policies or insufficient security measures, hence diminishing user trust.

The incorporation of mHealth applications into current healthcare systems poses an additional obstacle. Numerous applications function independently, lacking seamless integration with electronic health records (EHRs) or healthcare providers’ systems [2,3]. This deficiency in interoperability constrains their effectiveness for both patients and professionals. Patients may be required to manually enter data into applications that do not synchronize automatically with other platforms, so imposing additional hassles and diminishing participation [5,6]. The expense is a persistent concern for patients while utilizing mHealth applications. Some applications are complimentary, while others necessitate subscription fees or in-app purchases that may be financially prohibitive for certain users. Concealed expenses in ostensibly free applications may also discourage ongoing utilization. Moreover, the necessity for dependable internet access and contemporary mobile devices imposes an indirect financial strain on users in low-income environments [1].

The regulatory landscape for mHealth applications is fragmented and ambiguous in numerous areas. Patients frequently inquire about the responsibility for their treatment while utilising these tools specifically, whether it lies with the app creators or the healthcare practitioners [1,5]. Ethical issues emerge about informed permission and data ownership, as numerous users remain oblivious to the methods of data collection, storage, or sharing [5]. Patient motivation is essential for the uptake of mHealth applications. Numerous individuals lack the motivation to consistently utilize these technologies, particularly if they feel negligible quick advantages [2]. Time restrictions compound this issue; demanding schedules hinder patients’ ability to allocate time for learning new technology or consistently inputting data into applications [2].

Dependable technological infrastructure is crucial for the efficient utilization of mHealth applications, although it is frequently deficient in distant or underserved regions. Numerous rural and remote regions are deficient in the requisite infrastructure for high-speed internet, which is essential for uninterrupted video consultations [3]. Inadequate internet connectivity, obsolete devices, and compatibility challenges with specific operating systems impede access to these tools for numerous patients [5]. Failure to solve these infrastructural deficiencies restricts the accessibility of mHealth applications.

### 1.1. Sehaty Application

The Sehaty application, meaning “My Health” in English, is a comprehensive digital health platform created by the Saudi Ministry of Health (MoH). Initiated as a component of Saudi Arabia’s digital health transition, Sehaty functions as an all-encompassing healthcare management instrument for citizens and residents within the Kingdom. The Sehaty application in Saudi Arabia illustrates the capacity of mobile health applications to revolutionize healthcare access and provision. Sehaty aims to enhance the patient experience by offering a comprehensive suite of services, including the scheduling of medical appointments, access to teleconsultations, and prescription management. The application enables users to access medical data, obtain immunization updates, and monitor essential health metrics, such as step counts and heart rate, thus promoting a holistic approach to health management [7].

The application has achieved considerable popularity, boasting over 24 million users, which constitutes roughly 68.5% of Saudi Arabia’s population [8]. Throughout the COVID-19 pandemic, Sehaty significantly contributed to the national response by coordinating over 24 million testing appointments and administering more than 61 million vaccination doses [8]. The Sehaty app has emerged as a fundamental component of healthcare provision in Saudi Arabia, including vital services such virtual health consultations, follow-ups, and assessments. This platform, required for usage by all Saudi citizens and visitors, is essential for facilitating healthcare access in accordance with the digital transformation objectives of Saudi Vision 2030. Comprehending the barriers to the usage of the Sehaty app is essential for improving its efficacy and guaranteeing equitable access to healthcare services. This study enhances the app’s functionality by tackling technical, usability, and accessibility difficulties, hence reinforcing its significance as an essential tool in the healthcare system for millions of users throughout the Kingdom.

### 1.2. Study Aims and Objectives

This study aims to explore healthcare practitioners’ perceptions of virtual physicians and mHealth applications, identify the barriers to their adoption, and provide insights into how these technologies can be optimized to enhance patient care and improve the integration of digital health tools within existing healthcare systems.

- To assess healthcare practitioners’ perceptions of the usability and effectiveness of mHealth applications, with a focus on the Sehaty app.
- To identify the key barriers to the adoption of mHealth applications, including usability, privacy concerns, and integration challenges.
- In enhancing patient care, particularly in chronic disease management.
- To provide practical recommendations for improving the accessibility, usability, and integration of mHealth technologies into existing healthcare systems.

## 2. Methods

This study employed a cross-sectional design targeting patients who had used the Sehaty app in Saudi Arabia. The cross-sectional method enabled a temporal evaluation of the barriers faced by app users at a specific time.

### 2.1. Population and Sampling

The research focused on primary healthcare providers in Saudi Arabia utilising the Sehaty application, with an estimated population of 76,329 individuals [9]. This group comprises practitioners from several professions at primary healthcare centres (PHCs) throughout various parts of the country. Comprehending the experiences and barriers faced by these practitioners in utilising Sehaty is essential for assessing the system’s efficacy and usability in standard clinical practice.

Due to time limitations and accessibility issues, convenience sampling was chosen as the principal recruiting approach. This non-probability sampling method facilitated the quick gathering of responses from persons who were accessible and willing to engage. Nevertheless, acknowledging the possibility of bias in convenience sampling, various measures were employed to improve sample variety and representativeness. The sample size was augmented to 409 people to guarantee a wider spread of replies. Secondly, stratified sampling was employed to proportionately reflect various professions, geographic regions, and degrees of experience within PHCs. Third, precise inclusion and exclusion criteria were formulated to guarantee congruence between the sample and the target population. A multi-method recruitment strategy, encompassing online surveys, in-person outreach, and workplace collaborations, was utilised to engage a broader population. Finally, demographic distributions were observed throughout data collecting, and further initiatives were implemented to involve under-represented groups as necessary. These methods combined reduced bias and enhanced the overall quality of the dataset.

Cochran’s formula was utilised to ascertain the necessary sample size, employing a 95% confidence level, a 5% margin of error, and an estimated 50% response distribution (*p* = 0.5) to accommodate maximum variability. Considering the overall population of 76,329 practitioners, the initially computed sample size was modified using the finite population adjustment, yielding a requisite minimum sample size of 382 responses. The survey finally garnered 409 valid replies, resulting in a response rate of 107.07%, surpassing the minimal sample size requirement. The robust response rate indicates significant participant engagement, hence enhancing the credibility of the study results despite the intrinsic limits of convenience sampling.

### 2.2. Data Collection Instrument

Data were collected via a standardized questionnaire aimed at examining the barriers to the adoption of mobile health applications, particularly the Sehaty app (see Supplementary File S1). The questionnaire was formulated based on ten primary characteristics recognized in the literature as potential barriers, including Technical Barriers, Usability Barriers, Support and Training, Accessibility Barriers, Privacy and Security Barriers, Communication and Interaction Barriers, Functionality Barriers, User Satisfaction Barriers, Cost and Accessibility Barriers, and Time and Productivity Barriers. Each variable was operationalized using 4–6 items, culminating in a total of 49 items intended to comprehensively capture users’ perceptions and experiences with the application.

The questionnaire comprised four primary sections. The initial component contained a letter of assurance detailing the study’s objective and nature, ethical considerations, and participant rights. It underscored the voluntary aspect of participation, the confidentiality of responses, and the procedures implemented to guarantee data security and anonymization. The second half concentrated on demographic inquiries to collect data regarding participants’ age, gender, occupation, years of experience in their profession, and familiarity with mobile health applications. The final segment consisted of 49 items corresponding to the 10 previously described variables, each targeting specific barriers to the adoption and utilization of mobile health applications.

The questionnaire included a fourth section designed to assess the influence of physician chatbots, like ChatGPT, on patient healthcare as perceived by healthcare practitioners. This section included 20 Likert-scale items, from “Strongly Agree” to “Strongly Disagree”, enabling participants to articulate their perspectives on the potential advantages and barriers of incorporating AI-based physician tools in clinical environments. The items addressed patient engagement, care quality, practitioner workload reduction, and the effectiveness of virtual physicians in enhancing patient care.

The questionnaire aimed to deliver a thorough assessment of participants’ interaction with the Sehaty app, documenting their views on its usability, utility, and the difficulties they faced. This systematic method guaranteed the acquisition of comprehensive data that illustrate the extent to which these barriers affected the adoption and use of the application.

### 2.3. Data Collection Process

The data collection was conducted online, targeting users of the Sehaty application in Saudi Arabia. Participants were provided with a link to the questionnaire, and their responses were recorded online. The online data collection strategy was chosen for its convenience for participants and its efficacy in engaging a broad audience of app users. The questionnaire was distributed over a two-month period, commencing on 11 August 2024. The questionnaire was distributed to healthcare practitioners via an online survey platform to facilitate accessibility and response efficiency. To enhance participant engagement and response rates, two reminders were dispatched: one during the third week and another during the sixth week of the data collection period. These reminders aimed to promote participation and guarantee that the sample encompassed a diverse array of practitioners, thereby improving the reliability and comprehensiveness of the collected data.

### 2.4. Data Analysis

Data analysis was performed using SPSS version 29. Descriptive statistics, including frequencies, percentages, and averages, were calculated for each of the 45 items to encapsulate participants’ replies. This investigation offered insights into the incidence and severity of perceived barriers to the use of mobile health applications. The internal consistency of the questionnaire was assessed using Cronbach’s alpha to evaluate the reliability of the overall instrument and the 10 variables, each representing unique aspects of barriers to the utilization of the Sehaty app. Additionally, inferential statistics were conducted to ascertain whether significant disparities existed in participants’ responses among different demographic categories. Correlation studies were performed to investigate the links among the primary variables, providing enhanced understanding of the interrelated barriers to mobile health application utilization.

The R software (Version 4.3.0) was utilized to illustrate the correlation among the ten principal variables (themes). Correlation analysis revealed insights into the relationships among several barriers to mobile health app utilization, highlighting potential interdependencies and areas requiring additional action.

### 2.5. Data Collection Validity and Reliability

A pilot study was conducted with a small group of seven healthcare practitioners, including two physicians, four nurses, and one administrator, to ensure the validity and appropriateness of the questionnaire. This pilot study aimed to evaluate the clarity, readability, and relevance of the questionnaire items concerning the study’s objectives. Participant feedback was utilized to identify ambiguities, inconsistencies, or unclear instructions that could impede respondents’ understanding or compromise the accuracy of their responses. The pilot study yielded important insights regarding the questionnaire’s overall flow, facilitating the refinement of specific item wording and the adjustment of response scales to better reflect participants’ experiences and perspectives. This pilot study led to several modifications aimed at enhancing the questionnaire’s construct validity, ensuring accurate identification of barriers to the adoption of mobile health applications, specifically the Sehaty app, and its appropriateness for the target population.

The questionnaire’s reliability was evaluated through Cronbach’s alpha for each primary variable, revealing that all scales exhibited acceptable to excellent internal consistency. The variables demonstrating the highest reliability included Cost and Resource Barriers (α = 0.91), Time and Productivity Barriers (α = 0.89), and Impact of Physician chatbot (α = 0.95), reflecting robust internal consistency in these domains. Additional variables, including Technical Barriers (α = 0.80) and Data Management and Security Barriers (α = 0.85), demonstrated reliable internal consistency as well. The questionnaire demonstrated excellent overall reliability, evidenced by a Cronbach’s alpha of 0.95, indicating that the instrument yields consistent measurements across all assessed barriers and constructs (See Table 1).

## 3. Result

### 3.1. Demographic Characteristics of Participants

**Figure 1:**
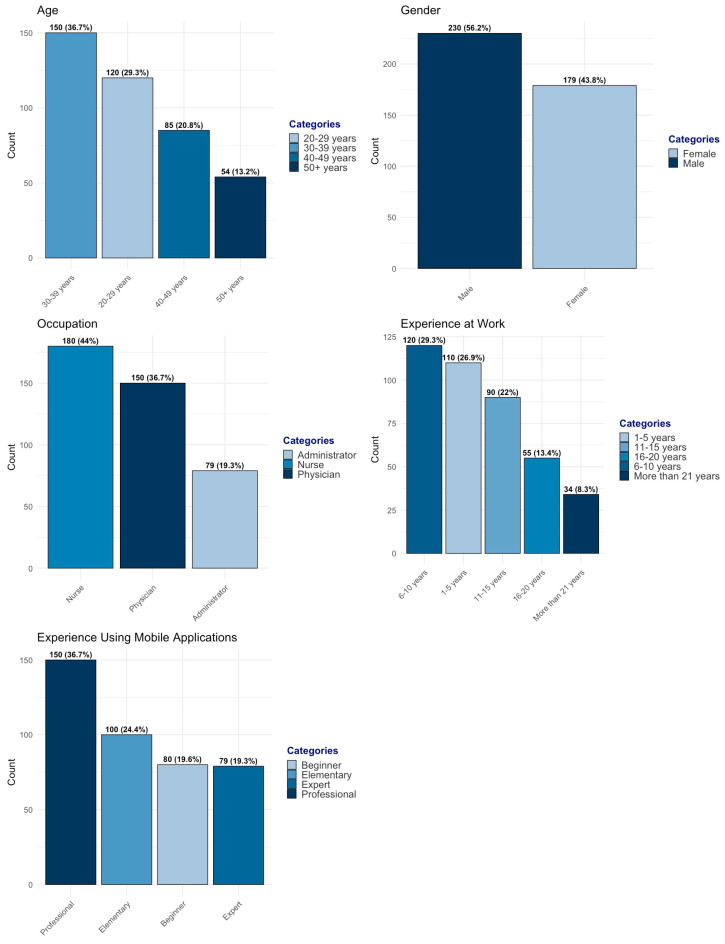
Practitioners’ demographic distribution.

The study included 409 primary healthcare practitioners, representing a diverse range of ages, professional roles, work experience levels, and mobile application proficiency. The age distribution shows that the majority of participants were in the 30–39 years group (36.7%), followed by 20–29 years (29.3%), while 40–49 years (20.8%) and 50 years and above (13.2%) comprised smaller proportions (see Figure 1). The gender distribution was nearly balanced, with 230 female participants (56.2%) and 179 male participants (43.8%).

Regarding professional roles, nurses formed the largest group (44.0%), followed by physicians (36.7%) and administrators (19.3%). The work experience distribution was well spread across different levels, with 6–10 years (29.3%) and 1–5 years (26.9%) being the most common, followed by 11–15 years (22.0%), 16–20 years (13.4%), and more than 21 years (8.3%). Participants also varied in their experience with mobile applications, with 36.7% identifying as professionals, 24.4% as elementary users, 19.6% as novices, and 19.3% as experts.

To enhance diversity and representativeness, a stratified approach was incorporated within the convenience sampling method. This ensured proportional representation across key professional groups, work experience levels, and mobile application proficiency categories. The sample composition aligns with workforce distribution trends in Saudi Arabian primary healthcare centers, reinforcing its relevance to real-world settings. By ensuring balanced participation across demographic subgroups, the study minimizes sampling bias and strengthens the applicability of findings to healthcare practitioners using the Sehaty application.

**Figure 2:**
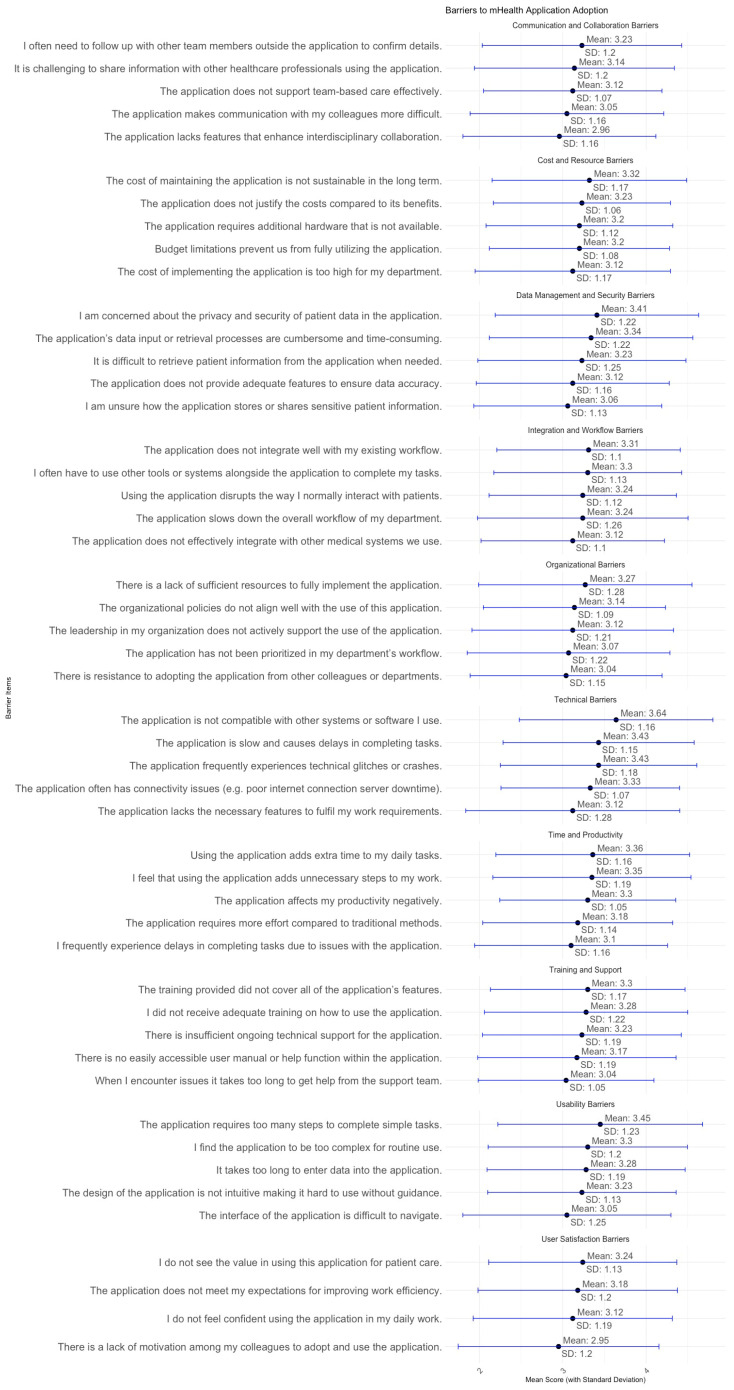
Mean score and SD for Practitioners’ perception towards the implementation of the mHealth services.

Figure 2 identified mean score and SD of key barriers affecting the adoption and use of the Sehaty application among primary healthcare practitioners. The findings highlight challenges across multiple dimensions, particularly technical performance, usability, training, workflow integration, data security, organizational support, communication, cost, productivity, and adoption barriers. These factors influence the system’s usability and effectiveness, requiring targeted improvements to enhance engagement and efficiency.

Compatibility concerns (Mean = 3.64, High) emerged as a significant barrier, highlighting challenges in integrating Sehaty with current healthcare systems. Likewise, sluggish system performance (Mean = 3.43, Moderate) and recurrent malfunctions or crashes (Mean = 3.43, Moderate) impeded workflow efficiency. Connectivity issues, such as server outages and internet instability (Mean = 3.33, Moderate), adversely affected system usability. These technical issues underscore the necessity for infrastructure improvements, especially in system stability, velocity, and interoperability. Usability issues were significant, with participants reporting intricate navigation (Mean = 3.05, Moderate) and an excessive number of steps needed to accomplish activities (Mean = 3.45, Moderate), highlighting the necessity for a more intuitive and simplified interface.

Barriers with training and support were notably influential, as participants indicated inadequate training (Mean = 3.28, Moderate) and restricted continuous technical assistance (Mean = 3.23, Moderate). The absence of accessible user manuals (Mean = 3.17, Moderate) and delays in obtaining assistance (Mean = 3.04, Moderate) indicate the need for organised training initiatives and improved user support systems. Challenges in workflow integration were apparent, as practitioners reported that the application disrupts established workflows (Mean = 3.24, Moderate) and lacks effective integration with other systems (Mean = 3.12, Moderate), frequently necessitating reliance on external tools (Mean = 3.30, Moderate) to accomplish tasks.

Concerns over data privacy and security (Mean = 3.41, Moderate) were significant, indicating ambiguity about the storage and sharing of patient data (Mean = 3.06, Moderate). Moreover, inefficient data entry and retrieval methods (Mean = 3.34, Moderate) revealed shortcomings in health information management, emphasising the necessity for more secure and user-friendly data management protocols. Organisational policies (Mean = 3.14, Moderate) and leadership support (Mean = 3.12, Moderate) were assessed as moderate, indicating that although some institutional support is present, more strategic leadership and policy alignment are necessary to promote wider adoption.

Concerns regarding productivity were pronounced, as users perceived the program to introduce superfluous steps to daily chores (Mean = 3.35, Moderate) and adversely affect productivity (Mean = 3.30, Moderate). These findings underscore the necessity for workflow optimisation and enhancements in efficiency. Conversely, colleague motivation for adoption (Mean = 2.95, Low) was among the least significant barriers, indicating that peer participation is not a principal impediment. Nonetheless, discontent persisted, as several participants felt the system failed to fulfil expectations for enhancing efficiency (Mean = 3.18, Moderate).

**Figure 3:**
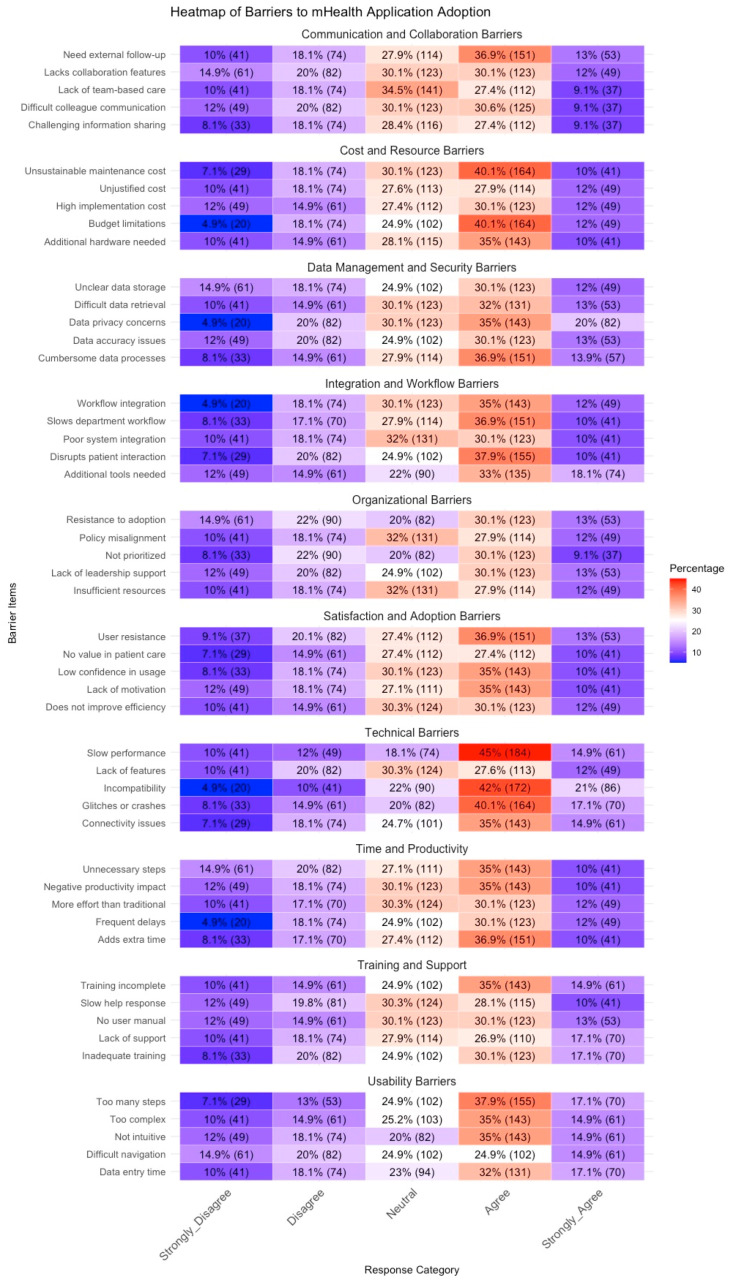
Likert scale responses of practitioners’ perception towards the implementation of the mHealth service.

Based on the heatmap (see Figure 3), the main barriers to mHealth application adoption are observed in Technical Barriers, Usability Barriers, and Time/Productivity Barriers, where a significant proportion of participants reported agreement (ratings of 2 and 1). Notably, slow performance (47.6% agreement), unnecessary steps (45%), and frequent delays (42%) were among the most critical challenges. Similarly, usability concerns, such as data entry complexity (42%) and non-intuitive navigation (40%), were frequently cited as obstacles. These findings suggest that users experience significant inefficiencies and usability frustrations that impact their overall satisfaction with the application.

Furthermore, Organizational Barriers and Communication Barriers also emerged as important constraints. A substantial proportion of participants reported insufficient resources (40%), lack of leadership support (38%), and poor integration with existing workflows (35%) as major concerns. Additionally, difficult colleague communication (37%) and challenges in sharing information (35%) indicate that the application does not facilitate effective teamwork. Privacy and security barriers were less severe in comparison but still showed moderate agreement, particularly regarding unclear data storage and access concerns (33%). Overall, these findings highlight the need for improvements in system performance, usability, and organizational support to enhance the successful adoption of mHealth applications.

### 3.2. Inferential Statistics

#### 3.2.1. Differences Tests

The ANOVA results in Table 2 indicate that age, occupation, and work experience do not significantly influence perceived barriers to mHealth adoption, as most *p*-values exceed 0.05. While Integration and Workflow Barriers (F = 2.343, *p* = 0.073) and Cost and Resource Barriers (F = 2.188, *p* = 0.089) show slightly higher F-values for age, these associations remain statistically non-significant. Similarly, Organizational Barriers (F = 2.467, *p* = 0.086) and Communication Barriers (F = 2.040, *p* = 0.131) suggest a potential influence of occupation but do not reach significance. These findings indicate that general demographic characteristics may not be strong determinants of perceived challenges in mHealth adoption.

In contrast, experience using mobile applications significantly impacts Communication Barriers (F = 2.683, *p* = 0.046), suggesting that users with less experience may encounter greater communication-related challenges when adopting mHealth solutions. Additionally, Integration and Workflow Barriers approach significance (F = 2.237, *p* = 0.083), indicating that digital literacy and familiarity with mobile technologies might influence perceptions of workflow integration. These findings highlight the importance of user experience and digital proficiency in shaping mHealth adoption barriers, suggesting that targeted training programs and usability enhancements may help mitigate communication and integration-related challenges.

The *t*-test results in Table 3 indicate that gender differences in perceived barriers to mHealth adoption are largely non-significant, with most *p*-values exceeding 0.05. However, Communication Barriers (t = 1.908, *p* = 0.057) and Time and Productivity Barriers (t = −1.740, *p* = 0.083) show marginal differences, suggesting that females may experience slightly greater communication challenges, while males report slightly higher productivity-related constraints. Although these differences do not reach statistical significance, they highlight potential gender-based variations in user experiences. These findings suggest that while gender-inclusive strategies are generally sufficient, targeted interventions aimed at improving communication support and time management features in mHealth applications may enhance usability for different gender groups.

#### 3.2.2. Correlation Tests

**Figure 4:**
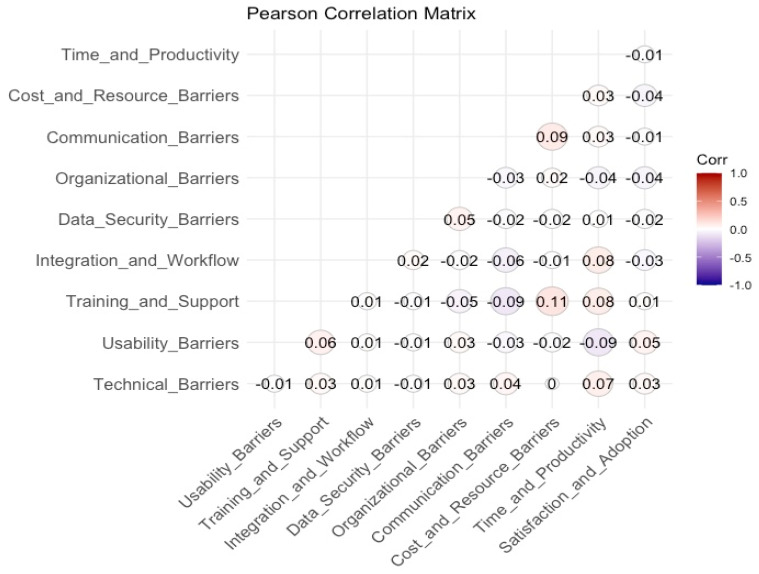
Pearson correlation matrix of main barriers in mHealth application.

The Pearson correlation matrix presented in Figure 4 illustrates the interrelationships among the key barriers affecting the adoption and usability of mHealth applications. The visualization employs a correlation heatmap where the size and colour intensity of the circles denote the strength and direction of the relationships. Positive correlations are depicted in shades of red, while negative correlations appear in shades of blue, with stronger hues indicating higher correlation values. The correlation coefficients range from −1 (indicating a perfect negative relationship) to 1 (indicating a perfect positive relationship), whereas values near zero suggest minimal or no linear association between the variables.

The analysis reveals that most of the examined barriers exhibit weak correlations, suggesting that these factors largely operate independently rather than being strongly interlinked. The strongest observed correlation is between Training and Support and Satisfaction and Adoption (r = 0.11), indicating that higher levels of training and support may be associated with improved satisfaction and adoption of mHealth applications. Additionally, Communication Barriers demonstrates weak positive associations with Satisfaction and Adoption (r = 0.09) and Cost and Resource Barriers (r = 0.09), suggesting that challenges in communication and resource constraints may slightly impact user satisfaction. Similarly, Technical Barriers exhibits a weak positive relationship with Satisfaction and Adoption (r = 0.07), implying that technical challenges may marginally influence adoption decisions.

Conversely, Organizational Barriers and Integration and Workflow Barriers display negligible correlations with other factors, reinforcing the idea that these barriers function independently within the mHealth adoption landscape. Data Security Barriers, often cited as a critical concern in digital health, shows minimal associations with other constructs, with its highest correlation being r = 0.05 with Training and Support. Time and Productivity Barriers exhibits near-zero correlations with most variables, indicating that perceived time constraints and productivity concerns do not significantly interact with other barriers.

Overall, the weak correlation values suggest that these barriers to mHealth adoption are distinct rather than highly interconnected. This finding underscores the necessity for targeted interventions that address specific barriers rather than a generalized approach. Efforts to improve training and support mechanisms may have the most noticeable impact on increasing user satisfaction and adoption, whereas addressing communication and technical challenges could contribute modestly to enhancing user experiences.

## 4. Discussion

This study underscores critical barriers to the adoption and usability of the Sehaty application among primary healthcare practitioners, with technical inefficiencies, usability challenges, and productivity concerns emerging as the most significant impediments. Compatibility issues (Mean = 3.64) and sluggish system performance (Mean = 3.43) were particularly pronounced, reinforcing the need for infrastructure enhancements to improve system speed, reliability, and interoperability. These findings align with prior research emphasizing that technical inefficiencies, particularly poor integration with existing healthcare systems, significantly hinder mHealth adoption [10,11]. Similarly, the study identified substantial usability concerns, with participants reporting complex navigation (Mean = 3.05) and excessive task steps (Mean = 3.45), both of which contribute to workflow inefficiencies. These results corroborate earlier studies that highlight the role of intuitive interface design in ensuring sustained user engagement [11,12]. The persistence of these challenges suggests that mHealth developers must prioritize user-centered design principles to enhance accessibility and efficiency.

Furthermore, inadequate training and technical support emerged as key obstacles, with participants indicating insufficient training (Mean = 3.28) and limited access to ongoing technical assistance (Mean = 3.23). These findings are consistent with previous research demonstrating that digital literacy and structured training programs play a pivotal role in mHealth usability [13]. Notably, the study found that integration and workflow barriers were substantial, with practitioners reporting that Sehaty disrupts existing workflows (Mean = 3.24) and lacks effective interoperability with other healthcare systems (Mean = 3.12). These findings echo concerns raised in Labrique, Wadhwani [14], which emphasized the challenges of aligning digital health systems with established clinical practices. Without seamless integration, healthcare providers may resort to parallel processes, undermining the intended efficiencies of digital transformation.

Concerns surrounding data security and privacy were also evident, with participants expressing uncertainty regarding patient data storage (Mean = 3.06) and difficulties in data retrieval (Mean = 3.34). These concerns align with prior studies that emphasize the centrality of data security in shaping user trust and engagement with digital health platforms [15,16]. Compared to studies in lower-resource settings, where privacy concerns are often exacerbated by weak regulatory frameworks [17], the moderate level of concern observed in this study suggests that while regulatory mechanisms in Saudi Arabia may be more developed, there remains a need for enhanced transparency regarding data governance and security protocols.

Interestingly, demographic variables such as age, occupation, and work experience did not significantly influence perceived barriers to mHealth adoption. This finding contrasts with previous studies indicating that older users and those with limited technological proficiency tend to report greater difficulties in adopting digital health tools [18]. However, experience with mobile applications significantly influenced perceptions of communication barriers (F = 2.683, *p* = 0.046), suggesting that digital literacy remains a crucial determinant of user engagement. This finding aligns with Khamaj and Ali [19], who demonstrated that individuals with greater digital proficiency encounter fewer usability challenges when interacting with mHealth applications. These insights reinforce the importance of targeted training programs to bridge the digital divide among healthcare practitioners.

In contrast to findings from developed countries, where interoperability and technical refinements are the primary challenges to mHealth adoption [20], this study suggests that while technical limitations persist, they are compounded by workflow inefficiencies and inadequate user support mechanisms. In lower-resource settings, barriers such as limited internet access and device shortages are predominant [21,22], whereas in the context of this study, the focus shifts toward optimizing system usability and enhancing productivity. These disparities underscore the necessity of tailoring mHealth strategies to regional needs, as the barriers to adoption are context-dependent.

Overall, the findings highlight the urgent need for a multi-pronged approach to address barriers to mHealth adoption. Prioritizing user-centered interface design, strengthening training programs, improving system integration, and enhancing organizational support structures are critical to fostering widespread adoption and sustained engagement. These recommendations align with previous research advocating for a holistic approach to digital health implementation, emphasizing that technological advancements must be complemented by policy reforms and end-user engagement strategies to maximize impact [23]. By addressing these key barriers, healthcare systems can leverage mHealth solutions more effectively to enhance care delivery, streamline workflows, and improve patient outcomes.

### 4.1. Practical Recommendations for Enhancing mHealth Accessibility, Usability, and Integration

To enhance the accessibility, usability, and integration of mHealth technologies within existing healthcare systems, several key recommendations emerge from this study’s findings and prior research. First, improving interoperability and establishing standardized protocols are essential to facilitate seamless integration between mHealth applications and Electronic Medical Record (EMR) systems. A unified data exchange framework would enable real-time access to patient information across healthcare platforms, thereby enhancing care coordination, reducing redundancies, and supporting evidence-based decision-making. Second, addressing usability challenges necessitates a user-centered design approach that prioritizes simplicity, intuitive navigation, and accessibility, particularly for populations with limited digital literacy. Complementary to this, educational initiatives and awareness campaigns are crucial in fostering cultural acceptance and trust in mHealth solutions, particularly in regions with historically low adoption rates.

Moreover, leveraging advanced data analytics in conjunction with mHealth applications presents an opportunity to optimize healthcare delivery by transforming raw data into actionable insights. The application of predictive modelling and artificial intelligence-driven analytics can facilitate early disease detection, personalized treatment recommendations, and real-time outbreak monitoring, thereby enhancing public health responses. Ensuring robust data security and privacy protections is equally critical, as the widespread adoption of mHealth depends on user confidence in data confidentiality. The implementation of advanced encryption methods, coupled with clear and transparent data governance policies, is necessary to align with regulatory requirements and mitigate security concerns. Finally, sustainable policy frameworks must be established to support long-term investments in mHealth infrastructure, secure funding mechanisms, and cross-sector collaborations among policymakers, technology developers, and healthcare providers. These strategic measures collectively contribute to fostering a more integrated, efficient, and patient-centric digital healthcare ecosystem.

This study makes a significant contribution to the literature by shifting the focus from patient-centered mHealth adoption challenges to the barriers encountered by healthcare practitioners. While previous research has extensively examined patient experiences with mHealth applications, limited attention has been given to the perspectives of healthcare professionals who are integral to the successful implementation of these technologies. By empirically identifying and quantifying key barriers, including technical, usability, training, integration, security, organizational, and productivity-related constraints, this study provides a structured and comprehensive framework for understanding the complexities of mHealth adoption in clinical practice. The findings highlight that while digital literacy levels significantly influence practitioners’ perceptions of these barriers, demographic factors such as age, occupation, and work experience do not exhibit a strong impact. Additionally, the study’s heatmap analysis and statistical evaluations reveal that technical performance, usability challenges, and workflow integration issues are among the most critical obstacles affecting mHealth adoption, underscoring the need for targeted improvements in system functionality and user experience.

A key theoretical insight from this study is the independence of mHealth adoption barriers, as demonstrated by weak correlations between different categories of challenges. This challenges conventional assumptions that addressing one barrier, such as usability, will automatically resolve others, such as workflow integration or communication constraints. Instead, the findings emphasize the need for multi-faceted, barrier-specific interventions that address each challenge independently. Furthermore, this study provides practical recommendations to enhance mHealth adoption, including improving interoperability with EMRs, implementing user-centered design approaches, strengthening data security measures, and developing structured training programs. These findings contribute to the global discourse on digital health transformation while offering context-specific insights tailored to the Sehaty application and the Saudi healthcare system. By providing empirical evidence and actionable recommendations, this study serves as a valuable resource for policymakers, healthcare administrators, and technology developers seeking to refine mHealth adoption strategies and enhance the integration of digital health solutions into primary healthcare settings.

### 4.2. Study Limitations, Strength and Recommendation for Future Research

This study has several limitations that warrant consideration. First, its focus on the Sehaty application within the Saudi Arabian healthcare system may constrain the generalisability of the findings to other mHealth platforms and international healthcare contexts. Future research should explore these barriers across diverse healthcare systems and digital health applications to enhance external validity. Second, while the quantitative survey approach provided a comprehensive assessment of practitioners’ perceptions, the absence of qualitative data, such as interviews or focus groups, limits the study’s ability to capture in-depth insights into the underlying reasons behind the identified barriers. Integrating qualitative methodologies in future research could offer a more nuanced understanding of user experiences and contextual challenges. Additionally, although efforts were made to ensure demographic diversity in the sample, the study does not explicitly report the response rate, which may affect the assessment of sample representativeness. Finally, while the discussion highlights the implications of the findings, a more detailed examination of the potential role of AI tools in mitigating mHealth adoption barriers would further enrich the analysis, providing valuable insights into the future integration of digital health solutions.

This study’s high response rate (107.07%), exceeding the required 382 participants with 409 valid responses, strengthens its generalisability and reliability. The broad participation across various healthcare professions, regions, and experience levels was facilitated by multi-method recruitment, including social media (WhatsApp), direct outreach, and workplace engagement. This approach helped mitigate selection and non-response biases, ensuring a more diverse and representative sample. Additionally, the integration of stratified sampling improved subgroup representation, while the anonymous online survey format minimized social desirability bias and enhanced data integrity. These methodological strengths reinforce the study’s validity and offer valuable insights for policymakers and healthcare stakeholders, optimizing the implementation of Sehaty in Saudi Arabia’s PHCs.

Future research should adopt a longitudinal approach to examine the sustained impact of virtual physician and mHealth on patient outcomes, including its effects on treatment adherence, patient engagement, and long-term health improvements. Comparative studies across diverse healthcare systems would further elucidate how contextual factors, such as digital infrastructure, regulatory frameworks, and workforce readiness, shape the adoption and efficacy of mHealth applications. Additionally, an in-depth investigation into patients’ perspectives on usability, trust, and satisfaction with virtual physicians is warranted. Understanding these dimensions will facilitate the development of more patient-centred digital health solutions, ensuring that mHealth innovations align with the needs and expectations of end-users while optimising healthcare delivery across different settings.

## 5. Conclusions

This study offers insights into healthcare practitioners’ perceptions of virtual physicians and physician chatbots, specifically regarding their impact on patient care enhancement. The findings indicate robust support for Physician chatbot aimed at enhancing patient access to healthcare, education, and chronic disease management; however, notable challenges persist in terms of integration and usability. Healthcare practitioners raised concerns regarding the integration of these technologies into current healthcare workflows and highlighted the necessity for sufficient training and support to facilitate successful adoption. Despite these concerns, virtual physicians are acknowledged as effective instruments for enhancing clinical decision-making, improving medication adherence, and enabling patients to manage their health more effectively. The findings emphasize the necessity of overcoming technical, organizational, and educational barriers to the adoption of virtual physicians. Future research should investigate the integration of AI tools into clinical practice, emphasizing the resolution of integration barriers and the assessment of their long-term impacts on patient care and outcomes.

## Figures and Tables

**Table 1:** Questionnaire reliability.

Main Variables	Number of Items	Cronbach’s Alpha
Technical Barriers	5	0.80
Usability Barriers	5	0.79
Integration and Workflow Barriers	5	0.75
Support and Training Barriers	5	0.90
Data Management and Security Barriers	4	0.85
Communication and Collaboration Barriers	6	0.81
Organizational Barriers	5	0.77
User Satisfaction Barriers	4	0.85
Cost and Resource Barriers	5	0.91
Time and Productivity Barriers	5	0.89
Impact of physician Chatbot	20	0.95
Entire questionnaire	65	0.95

**Table 2:** Influence of demographic and experience factors on barriers to mHealth application adoption: ANOVA test results.

Independent Variable	Dependent Variable	f Value	*p* Value
Age	Technical Barriers	0.101	0.96
	Usability Barriers	0.74	0.529
	Training and Support	0.021	0.996
	Integration and Workflow	2.343	0.073
	Data Security Barriers	0.272	0.846
	Organisational Barriers	0.171	0.916
	Communication Barriers	0.127	0.944
	Cost and Resource Barriers	2.188	0.089
	Time and Productivity	0.51	0.675
	User Satisfaction	0.943	0.42
Occupation	Technical Barriers	0.807	0.447
	Usability Barriers	0.516	0.597
	Training and Support	0.236	0.79
	Integration and Workflow	0.245	0.783
	Data Security Barriers	0.153	0.858
	Organisational Barriers	2.467	0.086
	Communication Barriers	2.04	0.131
	Cost and Resource Barriers	0.138	0.871
	Time and Productivity	1.449	0.236
	User Satisfaction	0.129	0.879
Experience at Work	Technical Barriers	0.562	0.69
	Usability Barriers	0.496	0.738
	Training and Support	0.605	0.659
	Integration and Workflow	1.289	0.274
	Data Security Barriers	0.448	0.774
	Organisational Barriers	0.13	0.971
	Communication Barriers	1.756	0.137
	Cost and Resource Barriers	0.634	0.638
	Time and Productivity	1.362	0.247
	User Satisfaction	0.646	0.63
Experience Using Mobile Applications	Technical Barriers	0.434	0.729
	Usability Barriers	1.336	0.262
	Training and Support	1.268	0.285
	Integration and Workflow	2.237	0.083
	Data Security Barriers	0.885	0.449
	Organisational Barriers	0.587	0.624
	Communication Barriers	2.683	0.046
	Cost and Resource Barriers	0.269	0.848
	Time and Productivity	1.131	0.336
	User Satisfaction	1.016	0.386

**Table 3:** Gender Differences in Barriers to mHealth Application Adoption: Results of Independent *t*-tests.

Dependent Variable	t Value	*p* Value	Mean Male	Mean Female
Technical Barriers	−0.987	0.324	3.41	3.359
Usability Barriers	1.501	0.134	3.226	3.309
Training and Support	−0.993	0.321	3.228	3.173
Integration and Workflow	−0.949	0.343	3.261	3.213
Data Security Barriers	1.04	0.299	3.213	3.269
Organisational Barriers	−0.181	0.856	3.131	3.121
Communication Barriers	1.908	0.057	3.057	3.155
Cost and Resource Barriers	0.862	0.389	3.17	3.214
Time and Productivity	−1.74	0.083	3.293	3.203
User Satisfaction	−0.62	0.536	3.134	3.097

## Data Availability

The study data are available from the corresponding author on reasonable request.

## Supplementary Materials

The following supporting information can be downloaded at: https://www.mdpi.com/article/10.3390/healthcare13050494/s1, File S1: Assurance letter for mHealth applications.
