# Supplementary material for: Healthcare Practitioners’ Perceptions of mHealth Application Barriers: Challenges to Adoption and Strategies for Enhancing Digital Health Integration
Source: Healthcare (Basel). 2025 Feb 25;13(5):494. doi: 10.3390/healthcare13050494 (PMC11899126; doi:10.3390/healthcare13050494)
Supplement: Supplementary file 1 [file healthcare-13-00494-s001.zip › healthcare-3432829-supplementary.pdf]

<u>Assurance letter for mHealth applications</u>

Examining Healthcare Practitioners' Perceptions of mHealth Applications, and Barriers to Adoption: Insights for Improving Patient Care and Digital Health Integration

You have received this questionnaire because you are currently working at healthcare sector. The questionnaire is part of a research study focused on the analysis of the effects of the introduction mHealth applications.

This questionnaire includes questions about your professional background, how much you come into contact with mHealth application, how effective you perceive it as and what the effects of its introduction may have been. A high response rate increases the validity of the study. Answering the survey is estimated to take between 10 to 20 minutes.

Voluntary participation: Participation in the study is voluntary, and you can refrain from participating at any time without having to explain why. There are no known benefits or risks to you from taking part in this study.

Confidentiality: The researcher will handle the data anonymously and with confidentiality. Access to primary reported material is restricted to the researcher only (your employer will not have access to your answers). Readers of reports from the study will not be able to identify individual responses.

Data security: The researcher will attempt to ensure the security of your data by extracting all the needed information from the paper questionnaires and filling the data into the appropriate software (e.g. SPSS & r). The researcher will lock the laptop with a strong password and install strong Internet protection software.

Publication of Findings: The outcomes of this research will be published online for the benefit of the academic and healthcare community. However, the published results will not include any information that could lead to the identification of individual participants.

Please do not hesitate to ask any questions.

Haitham Ali Alzghaibi
Saudi Arabia
Qassim University
Department of Health informatics
Email: halzghaibi@qu.edu.sa
Tel. 0555992566

Section one: Demographic information

1. **What is your age category?**
   - 18–24 years
   - 25–34 years
   - 35–44 years
   - 45–54 years
   - 55 years and above
2. **What is your gender?**
   - Male
   - Female
   - Prefer not to say
3. **What is your occupation?**
   - Physician
   - Nurse
   - Pharmacist
   - Allied Health Professional
   - Health Informatician
   - Medical Laboratory Professional
   - Public Health Professional
   - Administrator
   - Other (please specify): _______________
4. **How long have you been using mobile health applications?**
   - Less than 6 months
   - 6 months to 1 year
   - 1–3 years
   - 3–5 years
   - More than 5 years
5. **How many years of work experience do you have?**
   - Less than 1 year
   - 1–3 years

- 4–7 years
- 8–10 years
- More than 10 years

Section two: Barriers to the implementation of mHealth applications

Items	Strongly Disagree 1	Disagree 2	Neutral 3	Agree 4	Strongly Agree 5
The application frequently experiences technical glitches or crashes.					
The application is slow and causes delays in completing tasks.					
The application often has connectivity issues (e.g., poor internet connection, server downtime).					
The application is not compatible with other systems or software I use.					
The application lacks the necessary features to fulfill my work requirements.					
The interface of the application is difficult to navigate.					
The design of the application is not intuitive, making it hard to use without guidance.					
The application requires too many steps to complete simple tasks.					
It takes too long to enter data into the application.					
I find the application to be too complex for routine use.					
I did not receive adequate training on how to use the application.					
There is insufficient ongoing technical support for the application.					
There is no easily accessible user manual or help function within the application.					
The training provided did not cover all of the application's features.					
When I encounter issues, it takes too long to get help from the support team.					
The application does not integrate well with my existing workflow.					
Using the application disrupts the way I normally interact with patients.					
The application does not effectively integrate with other medical systems we use.					
The application slows down the overall workflow of my department.					
I often have to use other tools or systems alongside the application to complete my tasks.					
I am concerned about the privacy and security of patient data in the application.					
The application does not provide adequate features to ensure data accuracy.					

It is difficult to retrieve patient information from the application when needed.					
I am unsure how the application stores or shares sensitive patient information.					
The application’s data input or retrieval processes are cumbersome and time-consuming.					
The leadership in my organization does not actively support the use of the application.					
There is a lack of sufficient resources to fully implement the application.					
There is resistance to adopting the application from other colleagues or departments.					
The organizational policies do not align well with the use of this application.					
The application has not been prioritized in my department’s workflow.					
The application makes communication with my colleagues more difficult.					
The application does not support team-based care effectively.					
It is challenging to share information with other healthcare professionals using the application.					
The application lacks features that enhance interdisciplinary collaboration.					
I often need to follow up with other team members outside the application to confirm details.					
The cost of implementing the application is too high for my department.					
The application requires additional hardware that is not available.					
The cost of maintaining the application is not sustainable in the long term.					
Budget limitations prevent us from fully utilizing the application.					
The application does not justify the costs compared to its benefits.					
Using the application adds extra time to my daily tasks.					
The application requires more effort compared to traditional methods.					
The application affects my productivity negatively.					
I feel that using the application adds unnecessary steps to my work.					
I frequently experience delays in completing tasks due to issues with the application.					
I do not see the value in using this application for patient care.					
The application does not meet my expectations for improving work efficiency.					
I do not feel confident using the application in my daily work.					
There is a lack of motivation among my colleagues to adopt and use the application.					

Section three: the impact of AI tools (e.g. physician chatbot) to enhance the provision of healthcare

Items	Strongly Disagree	Disagree	Neutral	Agree	Strongly Agree
I believe virtual physicians (doctor chatbots) can improve the efficiency of patient triage in healthcare settings.					
Virtual physicians provide accurate and reliable medical information to support healthcare decisions.					
Using virtual physicians can reduce the workload of healthcare practitioners in routine consultations.					
Virtual physicians can improve access to healthcare services for patients in remote areas.					
I believe virtual physicians can enhance the overall quality of care in healthcare systems.					
I find virtual physicians easy to use and navigate in a healthcare environment.					
Training healthcare staff to work with virtual physicians is straightforward.					
Integrating virtual physicians into existing healthcare workflows is seamless.					
The interface of virtual physicians is intuitive and user-friendly for practitioners.					
I believe virtual physicians can be easily adapted for various medical specialties.					
AI chatbots enhance patient engagement by encouraging active participation in their healthcare.					
Virtual physicians improve patient adherence to treatment plans by providing regular reminders and follow-up support.					
AI chatbots are effective in addressing patients' questions about their treatment and medications.					
Patients feel more empowered in managing their health when using AI chatbots for consultation and support.					
AI chatbots can support chronic disease management by tracking symptoms and monitoring patient progress.					
Virtual physicians help patients schedule and manage routine check-ups and follow-ups effectively.					
AI chatbots reduce the communication gap between patients and healthcare providers, especially for chronic conditions.					
Virtual physicians can provide personalized health recommendations for chronic disease control.					

Patients with chronic conditions benefit from regular updates and tailored guidance offered by AI chatbots.					
Virtual physicians help patients set and achieve health-related goals, such as improving lifestyle or adhering to medication schedules.					

Section four: open ended questions

- In your opinion, what are the main barriers that healthcare practitioners and patients face when using mobile health (mHealth) applications in clinical practice?
- How do you perceive the role of AI-powered tools, such as virtual physicians or chatbots, in supporting healthcare delivery? What challenges or limitations do you foresee in their implementation?
- What improvements or changes do you believe are necessary to enhance the adoption and effectiveness of mHealth applications and AI tools in your field of work?
